# Molecular typing and mutational characterization of rectal neuroendocrine neoplasms

**DOI:** 10.1002/cam4.6281

**Published:** 2023-06-30

**Authors:** Xiaoling Duan, Man Zhao, Xiaolei Yin, Lili Mi, Jianfei Shi, Ning Li, Xin Han, Guangjie Han, Jinfeng Wang, Jiaojiao Hou, Fei Yin

**Affiliations:** ^1^ Department of Gastroenterology The Fourth Affiliated Hospital of Hebei Medical University Shijiazhuang China

**Keywords:** DDR mutant genes, genomic alterations, molecular typing, rectal neuroendocrine neoplasms, signaling pathway

## Abstract

**Background:**

Rectal neuroendocrine neoplasms (NENs) are rare neoplasms with limited understanding of its genomic alterations and molecular typing.

**Methods:**

The paraffin‐embedded tissue specimens of 38 patients with rectal NENs after surgery were subjected to whole gene sequencing (WGS), and mutation profilings were drawn to identify high‐frequency mutation genes, copy‐number variations (CNVs), tumor mutation burden (TMB), signal pathways, mutation signatures, DNA damage repair (DDR) genes, and molecular types. The differences of mutated genes and signaling pathways in different pathological grades and metastatic/non‐metastatic groups were compared. It helped to search for potential targets.

**Results:**

C > T and T > C transitions are the most common base substitutions in rectal NENs. DNA mismatch repair deficiency, DNA base modifications, smoking and exposure to ultraviolet light might play a role in the occurrence of rectal NENs. *DAXX*, *KMT2C*, *BCL2L1*, *LTK*, *MERTK*, *SPEN*, *PKN1*, *FAT3*, and *LRP2* mutations were found in only low‐grade rectal NETs, whereas *APC*, *TP53*, *NF1*, *SOX9*, and *BRCA1* mutations were common in high‐grade rectal NECs/MiNENs. These genes helped in distinguishing poorly‐differentiated or well‐differentiated rectal NENs. Alterations in P53, Wnt and TGFβ signaling pathways were more pronounced in rectal NECs and MiNENs. Alterations in Wnt, MAPK and PI3K/AKT signaling pathways promoted metastases. Rectal NENs were classified into two molecular subtypes by cluster analysis based on the mutant genes and signaling pathways combined with clinicopathological features. Patients with mutations in the *LRP2*, *DAXX*, and *PKN1* gene showed a trend of well‐differentiated and early‐stage tumors with less metastasis (*p* = 0.000).

**Conclusions:**

This study evaluated risk factors for regional lymphatic and/or distant metastases, identified high‐frequency mutated genes, mutation signatures, altered signaling pathways through NGS. Rectal NENs were divided into two molecular types. This helps to evaluate the likelihood of metastasis, formulate follow‐up strategies for patients and provide a target for future research on precision treatment of rectal NENs. PARP inhibitors, MEK inhibitors, mTOR/AKT/PI3K and Wnt signaling pathway inhibitors may be effective drugs for the treatment of metastatic rectal NENs.

## 1 INTRODUCTION

Neuroendocrine neoplasms (NENs), which have seen a rapid increase in incidence in recent years, can occur in whole‐body organs and tissues. According to the 2019 World Health Organization (WHO) classification of endocrine and neuroendocrine tumors (NETs), NENs are classified as well‐differentiated NETs, poorly differentiated neuroendocrine carcinomas (NECs) and mixed neuroendocrine‐non‐neuroendocrine neoplasms (MiNENs). According to the Ki‐67 index and mitotic rate (mitoses/2 mm^2^), NETs are classified as NET G1 (Ki‐67 index <3%, mitotic rate <2), NET G2 (Ki‐67 index 3%–20%, mitotic rate 2–20) and NET G3 (Ki‐67 index >20%, mitotic rate > 20%).^1^ Gastroenteropancreatic neuroendocrine neoplasms (GEP‐NENs) are the most common sites, accounting for about two‐thirds of all NENs. However, rectal NENs have the highest increase in incidence.^2^, ^3^ A retrospective multicenter study in China exploring the risk factors of lymph node metastasis in rectal NENs found that even small (<1 cm) and early NENs also have lymphatic metastases.^4^ The likelihood of lymphatic metastasis was 84.6% when the short diameter of the peri‐intestinal lymph node exceeded 5 mm.^5^ At present, no universally recognized prognostic evaluation system exists for rectal NENs. There were two studies on prognostic analysis of rectal NENs in US national cancer databases. One of them analyzed 777 patients with rectal NENs and found that age, sex, distant metastasis, surgical resection, and systematic treatment were the prognostic factors. Another study that analyzed 16,531 patients with rectal NENs found that age, insurance, pathological grade, tumor size, lymph node metastasis, operative margin status, and operative methods were the prognostic factors.^6^, ^7^ Patients with localized or regional rectal NENs could undergo endoscopic or surgical treatment, while patients with advanced metastatic diseases were given distinctive treatments because of different grades and stages. However, no unified effective standard was defined for choosing the best treatments, and the efficacy was limited. This indicates the need for individualized treatments.^8^ Recently, Scarpa et al. published a paper about the whole‐genome landscape of pancreatic NETs in *Nature*, which made an important contribution to the molecular progress of NENs.^9^ They found four signaling pathways including DNA damage repair, chromatin modification, altered telomere length and mTOR signaling, which were involved in the occurrence and development of pancreatic NETs. The efficacy of the mTOR inhibitor everolimus in pancreatic NETs was determined. Roy et al. found that nonfunctional pancreatic NETs with distant metastases contained alterations in *MEN1*, *ATRX*, *DAXX*, *TSC2*, and *DEPDC5* genes. Patients with primary pancreatic NETs and loss of *DAXX*, *ATRX*, *ARID1*, and/or *CDKN2A* had reduced survival times.^10^

Differences were present in the genetic background, responses to therapeutic drugs and prognosis between NETs and NECs. The key etiologically molecular drivers of NETs were genes such as *MEN1*, *DAXX*, and *ATRX*.^11^, ^12^ NECs displayed frequent inactivation of *RB1* and *TP53*, which were rare events in NETs. *Rb* and *KRAS* were promising predictors of NECs' response to platinum‐based chemotherapy.^13^

Most genomic profiling studies on NENs have focused on pancreatic and small intestine NETs.^11^, ^13^ Rectal NENs are highly heterogeneous tumors with multiple clinical outcomes and no prognostic biomarkers. Molecular characteristics, gene mutations and etiology of rectal NENs are less understood, leading to limited targeted therapies for patients. Genomic characterization of tumors is becoming increasingly common in clinical trials and standard of care. Owing to the extensive heterogeneity of rectal NENs, patients with the same grade and given the same therapy respond differently to the treatment. Our current understanding of the molecular characteristics of rectal NENs is insufficient for their clinical management, where the challenge is to resolve the current dilemma. There is an urgent need to explore the pathogenesis and molecular typing of rectal NENs from a comprehensive molecular genetic level and to classify patients more accurately. Based on different molecular types, we can find potential targets, specify more precise treatments and prevent recurrence and metastasis, to the greater benefit of patients.

## 2 MATERIALS AND METHODS

### 2.1 Patient and sample overview

**FIGURE 1:**
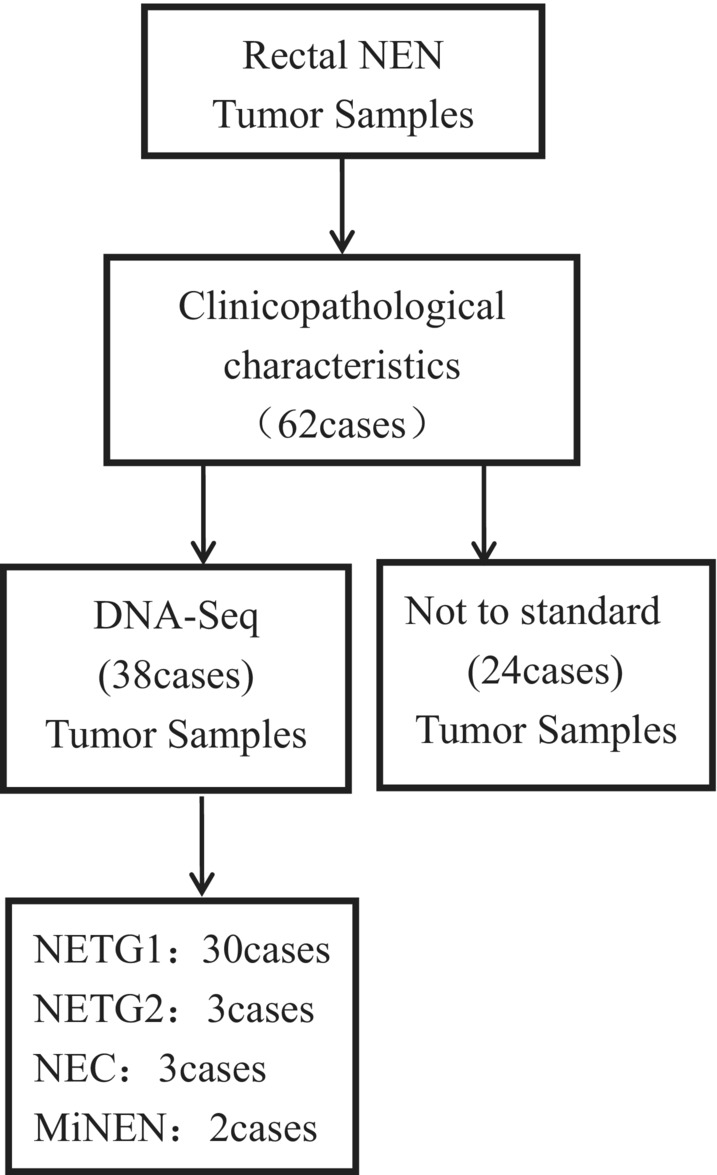
62 samples of rectal NEN. NETG1, neuroendocrine tumors grade 1; NETG2, neuroendocrine tumors grade 2; NEC, neuroendocrine carcinomas; MiNEN, mixed neuroendocrine‐non‐neuroendocrine neoplasm.

We retrospectively analyzed tumor samples from 62 patients with rectal NENs treated at the Fourth Hospital of Hebei Medical University between 2011 and 2019. Based on the 2019 WHO classification of endocrine and neuroendocrine tumors, we invited two pathologists to re‐identify the specimens. The neuroendocrine features were confirmed by hematoxylin–eosin and immunohistochemistry staining for neuroendocrine markers. The technical route was presented in the form of a graphical abstract. Among these 62 tumor samples, next‐generation sequencing (NGS) was performed on 38 specimens, and 24 samples with a small tumor volume were failure tested (Figure 1). The Ethics Committee of the Fourth Hospital of Hebei Medical University has reviewed and approved this study.

### 2.2 Next‐generation target sequencing

Thirty‐eight tumor tissues were obtained for NGS. The DNA was extracted using the QIAamp DNA FFPE Tissue Kit (Qiagen) and quantified using the Qubit dsDNA HS Assay Kit (Life Technologies, Carlsbad). The gDNA library was captured using a customized 671 gene individually synthesized 5′‐biotinylated DNA 120 bp oligonucleotides probe panel with xGen Hybridization and was quantified using the Qubit dsDNA HS Assay Kit. The captured libraries were sequenced on Illumina NovaSeq 6000 with 2 × 150 bp paired‐end reads, following the manufacturer's instructions (Illumina). The DNA‐seq reads were mapped to the hg19 reference sequence with BWA (version 0.7.12). The PCR duplicates were removed by Pi‐card (version 2.5.0) and recalibrated by the BaseRecalibrator tool from GATK (version 3.1.1). For tumor‐only samples, we first used a panel of normals in‐house to highlight somatic mutations. We also relied upon the public database of dbSNP build155, ExAC and 1000 Genomes as a germline source to remove the matching mutations that occur with at least minor allele frequency greater than 0.01.

### 2.3 Bioinformatic analyses

The raw sequencing data underwent stringent quality control of read depth and ratio of target capture. All types of genetic alterations, including single‐nucleotide variants (SNVs), short and long insertions and deletions (indels), copy number alterations (CNA) and gene rearrangement, were called using a suite of bioinformatics pipelines described in the supplemental online methods. The tumor mutation burden (TMB) score was calculated from cancer sequencing YS panel (CSYS) data for each sample by counting the number of somatic mutations, including coding SNVs and indels, per megabase (Mb) of the sequence examined. Known somatic mutations in the Catalog of Somatic Mutations in Cancer (COSMIC) and known germline polymorphisms in the U.S. Gene mutation spectrum were drawn by R package. We performed mutation spectrum and mutation signature analyses using NMF (version 0.22). Copy number variation (CNV) events were analyzed by GISTIC2. A KEGG database analysis was used to evaluate the pathway enrichment. Molecular subtypes were classified by K‐means clustering in SPSS (v 21.0). The heat map was drawn by R language with the heatmap package.

### 2.4 Statistical analysis

The relationship between metastatic/non‐metastatic diseases and clinicopathological characteristics was analyzed by the chi‐squared test. Fisher's exact test was used to analyze the relationship between differentially mutated genes and clinical characteristics. A two‐sided value of *p* < 0.05 was considered significant. All analyses were performed in R package and SPSS (v 21.0).

## 3 RESULTS

### 3.1 Patient clinical characteristics

Table 1 presents a summary of the patients' clinical information and tumor characteristics. The patients' ages ranged from 26 to 82 years, with a median of 54 years. The male‐to‐female ratio was 1.82:1. More patients presented with NETs (54, 87.1%) than NECs (6, 9.7%) and MiNENs (2, 3.2%). Patients with regional lymph node metastases and/or distant metastasis were present in 22 (35.5%) tumors. Regional lymph node metastases and/or distant metastasis of rectal NENs were related to tumor sizes (*p <* 0.001), tumor stages (*p <* 0.001) and tumor classification (*p <* 0.001) (Table 1).

**TABLE 1:** Analysis of clinicopathological features associated with metastasis of rectal NENs.

Factors	Total (n%)	Localized	Regional and/or distant	*p*
Gender				0.915
Male	40 (64.5%)	26 (41.9%)	14 (22.6%)	
Female	22 (35.5%)	14 (22.6%)	8 (12.9%)	
Age				0.758
≤55y	27 (43.5%)	18 (29.0%)	9 (14.5%)	
>55y	35 (56.5%)	22 (35.5%)	13 (21.0%)	
Tumor size				<0.001
≤1 cm	41 (70.7%)	37 (63.8%)	4 (6.9%)	
>1 cm	17 (29.3%)	3 (5.2%)	14 (24.1%)	
Tumor stage				<0.001
T1	36 (58.1%)	33 (53.2%)	3 (4.8%)	
T2–4	26 (41.9%)	7 (11.3%)	19 (30.6%)	
Metastasis				
Localized	40 (64.5%)	—	—	
Regional and/or distant	22 (35.5%)	—	—	
Tumor classification				<0.001
NETG1 + NETG2	54 (87.1%)	40 (64.5%)	14 (22.6%)	
NEC + MiNEN	8 (12.9%)	0	8 (12.9%)	
Distance from the edge of the anus				0.814
≤5 cm	31 (52.5%)	19 (32.2%)	12 (20.3%)	
>5 cm	28 (47.5%)	18 (30.5%)	10 (17.0%)	

### 3.2 Mutation signatures in rectal NENs

**FIGURE 2:**
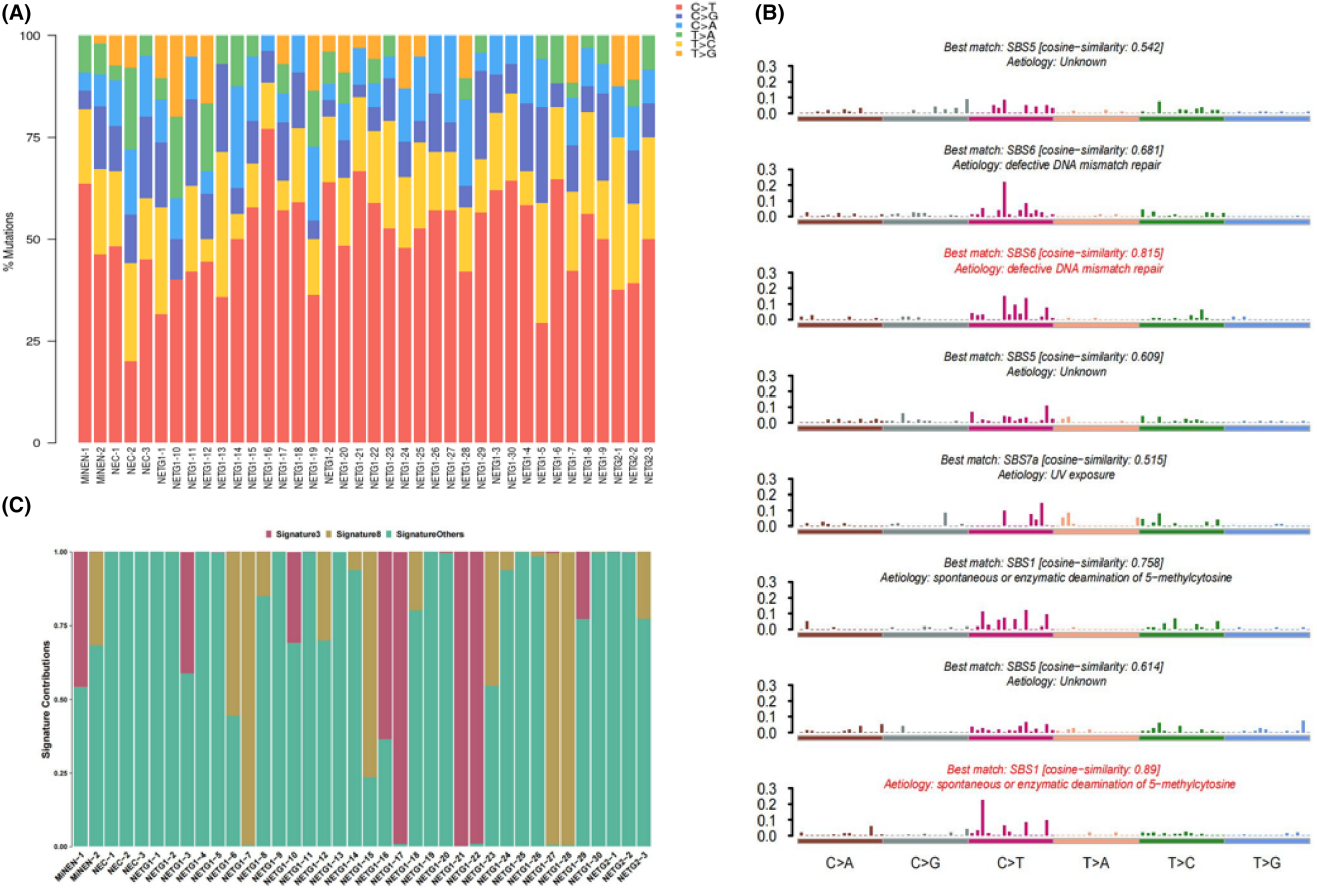
Mutation signatures in rectal NENs. (A) SNP mutation spectrum. X axis: Pathological grade; Y axis: the proportion of each mutation type. (B) Eight mutational signatures matched with COSMIC database. (C) The proportion of each mutation feature in different samples.

Numerous mutational processes occur when cells transition from a normal state to a stage of malignancy. These mutational signatures are partly responsible for the different etiologies. Various mathematical methods have been proposed to extract mutational features from the catalog of somatic mutations in cancer by using non‐negative matrix factorization (NMF). Profiles of single nucleotide mutation types were drawn through OmicStudio (Figure 2A). C > T and T > C transitions are the most common base substitutions in rectal NENs. To determine the contribution of these mutation characteristics to the etiology, we investigated mutational signatures by using the NMF algorithm. We identified eight mutational signatures (S1–S8) in the 38 rectal NENs (Figure 2B). All the signatures corresponded to mutation signatures in the COSMIC database of human cancer single base substitution (SBS) signatures. Compared with the COSMIC signatures, we found that S2 and S3 were related to DNA mismatch repair deficiency (SBS6); S6 and S8 to spontaneous or enzymatic deamination of 5‐methylcytosine (SBS1); S1, S4, and S7 to smoking (SBS5) and S5 to exposure to UV light (SBS7a). The similarity between S3 and SBS6 was 0.815, and the similarity between S8 and SBS1 was 0.89. The clustering analysis of NMF signatures displayed three distinct subtypes across the 38 rectal NENs patients, denoting NMF cluster S3, S8, and other signatures mixed with S1, S2, S4, S5, S6, and S7. The proportion of different mutational signatures in each patient was further analyzed (Figure 2C). Other signatures were predominant signatures in the two rectal MiNENs, three rectal NECs and three rectal NETG2. S3 and S8 also accounted for a certain proportion in rectal NETG1. Therefore, our results suggest that multiple factors such as DNA mismatch repair defects, DNA base modification, smoking, and exposure to ultraviolet light may contribute to the initiation of rectal NENs.

### 3.3 High frequency mutated genes

**FIGURE 3:**
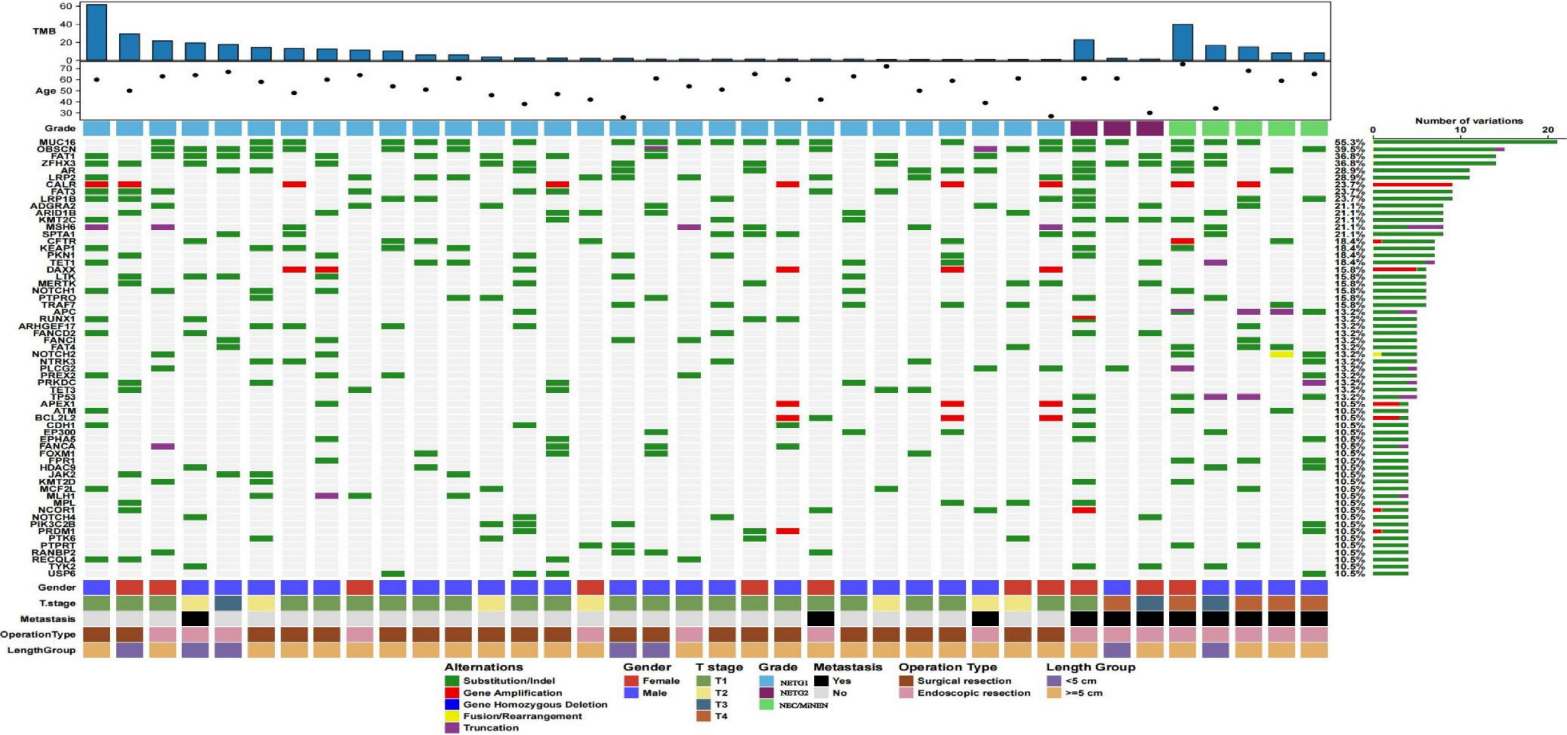
Mutation profiles of rectal NEN related genes:High frequency mutation genes are shown at the left. Mutant frequencies are shown on the right, TMBs are shown at the top, and associated clinicopathological characteristics for all 38 patients are shown at the bottom.

A total of 781 somatic SNVs, 83 short small indels, 42 gene amplifications, 34 gene truncations, 14 splice sites, 3 gene deletions, 2 gene rearrangements, and 1 gene fusion were identified. Genes with a mutation frequency of more than four were considered high‐frequency mutation genes. We searched for somatic alterations in 62 mutated genes of rectal NENs in the entire series of 38 tumor samples (Figure 3). The most recurrently altered genes were *MUC16* (21, 55.3%), *OBSCN* (15, 39.5%), *FAT1* (14, 36.8%), *ZFHX3* (14, 36.8%), *AR* (11, 28.9%) and *LRP2* (11, 28.9%).

**FIGURE 4:**
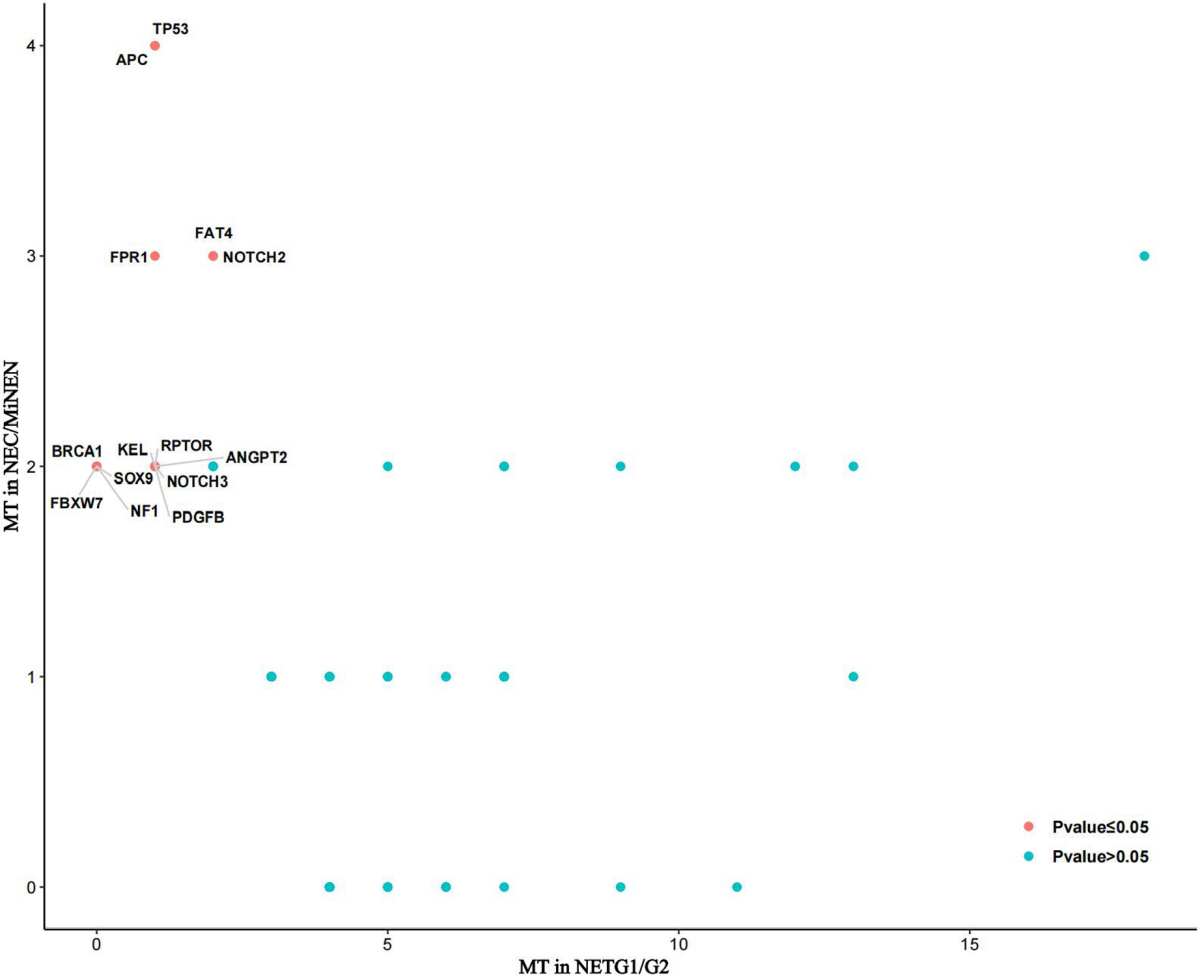
Scatter plots depicting the mutational frequencies (percentage of patients) between patients with rectal NETG1/NETG2 and NEC/MiNEN in our cohort. Each dot represents one gene, and dots are color coded according to the *p*‐values (−log10(P) uncorrected) shown in the legend. Statistics shown were derived from two‐sided Fisher's exact tests.

We conducted further exploration into these high‐frequency mutation genes and correlated these genes with the patients' clinical characteristics. Mutated genes were found in different grades of rectal NENs (Table S1). Among them, *APC*, *TP53*, *FPR1*, *FAT4*, *NOTCH2*, *BRCA1*, *FBXW7*, *NF1*, *SOX9*, *ANGPT2*, *KEL*, *NOTCH3*, *PDGFB*, and *RPTOR* were significantly different in different grades of rectal NENs (Figure 4). These genes in rectal NECs and MiNENs were more than those of rectal NETG1/G2. Owing to the small number of cases, any possible clinical significance needs to be verified by subsequent immunohistochemistry and cell experiments. Notably, mutations in *FANCD2* (5 cases), *RUNX1* (5 cases), *TET3* (5 cases), *DAXX* (6 cases), *LTK* (6 cases), *MERTK* (6 cases), *PKN1* (7 cases), *FAT3* (9 cases), and *LRP2* (11 cases) were not statistically different in different grades; however, these mutations were found in only NETG1 and NETG2, not in rectal NECs and MiNENs.

Significantly different mutant genes were also found in metastatic and non‐metastatic rectal NENs, including *TP53*, *TYK2*, *PDGFB*, and *APC*. These mutated genes were common in metastatic rectal NENs. The patients with the above mutant genes had a high metastatic risk (Table S2 and Figure S1).

### 3.4 Somatic CNVs in rectal NENs

Among the 38 cases of rectal NENs, GISTIC2 analysis was used to delineate significant CNV events, which revealed the top 20 significant amplifications and deletions (Figure S2). The figure shows significant amplifications of *MCL1*, *EPCAM*, *IRF2*, *NPM1*, *EED*, *KDM5A*, *NAB2*, *BCL2L2*, *APEX1*, *PRKACA*, and *PKN1* and deletions of *NRG2*, *FGF4*, *FAT3*, *FOXM1*, *FGF23*, *CHD4*, *FGF6*, *NTRK3*, and *MUC16*. The results show that the amplifications of oncogenes *CALR* (9 cases), *DAXX* (5 cases), *BCL2L1/2* (5 cases), *APEX1* (3 cases) and *NFKBIA* (3 cases) and deletions of tumor suppressor genes *CDKN2A*, *CDKN2B*, and *MTAP* were found in a single sample (Figure S3). No significant differences in clinicopathological features were observed between gene amplifications and deletions (Table S3).

### 3.5 DDR mutants

DNA damage repair (DDR) genes is a general term for a class of genes including *BRCA1*, *BRCA2*, *BLM*, *FANCA*, *TP53*, *RAD51C* and *MSH2*. DDR genes play an important part in the human DNA damage repair mechanism. Among the 38 patients with rectal NENs, DDR genes included *MSH6*(7), *FANCD2*(5), *ATM*(4), *PRKDC*(4), *MLH1*(4), *RECQL4*(4), *ATR*(3), *BRCA*(3), *DOT1L*(3), *ERCC5*(3), *MRE11*(3), *PARP1*(3), *RECQL4*(3), *RAD51C*(5), *BARD1*(2), *BLM*(2), *cCNE1*(2), *ERCC4*(2), *PALB2*(2), *TSC2*(2), *ZNF703*(2), *SLX4*(2), *CHD4*, *CDKN2A*, *CHD2*, *ATRX*, *CDK2*, *MSH3*, *CHEK2*, *EWSR1*, *NSD1*, and *SMARCA4* (Figure S4). A total of 81.6% (31/38) of rectal NENs had DDR mutants. The most significant mutant genes were *MSH6*, *FANCD2*, *ATM*, and *PRKDC*. Among the rectal NENs, we observed a significantly higher TMB in patients with mutated DDR genes than that in those without DDR genes (*p* = 0.029) (Figure S5). It is suggested that patients with rectal NENs harboring DDR mutations may benefit from PARP inhibitors.

### 3.6 TMB and clinicopathological features

**FIGURE 5:**
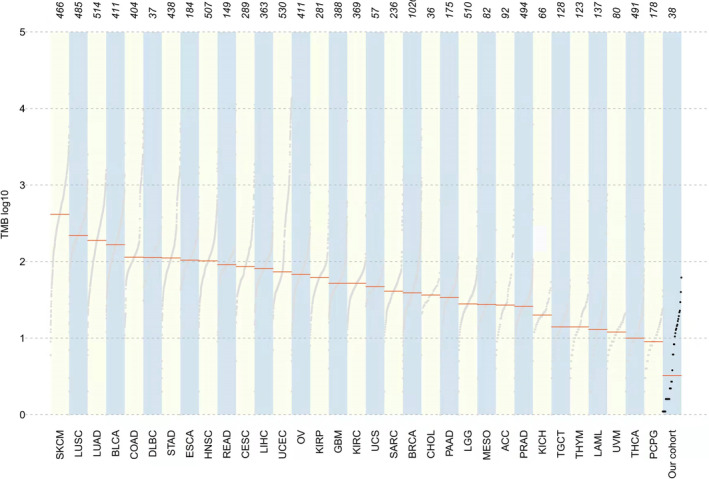
Landscape of tumor mutation burden (TMB) across the major tumor types; the median level of TMB for each tumor type was higher than rectal NEN of our cohort.SKCM, skin cutaneous melanoma; LUSC, lung squamous cell carcinoma; LUAD, lung adenocarcinoma; BLCA, bladder urothelial carcinoma; COAD, colon adenocarcinoma; DLBC, lymphoid neoplasm diffuse large B‐cell lymphoma; STAD, stomach adenocarcinoma; ESCA, esophageal carcinoma; HNSC, head and neck squamous cell carcinoma; READ, rectum adenocarcinoma; CESC, cervical squamous cell carcinoma and endocervical adenocarcinoma; LIHC, liver hepatocellular carcinoma; UCEC, uterine corpus endometrial carcinoma; OV, ovarian serous cystadenocarcinoma; KIRP, kidney renal papillary cell carcinoma; GBM, glioblastoma multiforme; KIRC, kidney renal clear cell carcinoma; UCS, uterine carcinosarcoma; SARC, sarcoma; BRCA, breast invasive carcinoma; CHOL, cholangio carcinoma; PAAD, pancreatic adenocarcinoma; LGG, brain lower grade glioma; MESO, mesothelioma; ACC, adrenocortical carcinoma; PRAD, prostate adenocarcinoma; KICH, kidney chromophobe; TGCT, testicular germ cell tumors; THYM, thymoma; LAML, acute myeloid leukemia; UVM, uveal melanoma; THCA thyroid carcinoma; PCPG pheochromocytoma and paraganglioma.

TMB is considered an effective biomarker for immunotherapy. It was estimated for 38 rectal NENs. The median TMB score was 9.87 (1.1–61.8) Muts/Mb, which was lower than that in other tumors of TCGA cohorts (Figure 5). A total of 47.4% of the samples had TMB ≥5 Muts/Mb. All these specimens were microsatellite stable (MSS) and negative in programmed death 1 ligand (PD‐L1). We further analyzed the relationship between TMB and patients' clinical pathological characteristics. High TMB was observed in groups of age > 55 years (*p* = 0.046), NEC and MiNENs (*p* = 0.040) (Table 2). TMB of NET G1 and NET G2 was significantly lower than that of NECs and MiNENs (8.6 vs. 17.7, *p* = 0.008) (Figure S6). No significant difference in TMB was found between metastatic and non‐metastatic rectal NENs. Older patients with rectal NECs and MiNENs may benefit from immunotherapy.

**TABLE 2:** Relationship between TMB and clinicopathological characteristics.

Factors	TMB(≤5)	TMB(>5)	*X* ^*2*^	*p*
Gender			0.023	0.880
Male	14	13		
Female	6	5		
Age			3.979	0.046*
≤55y	12	5		
>55y	8	13		
Tumor size			0.874	0.350
≤1 cm	13	9		
>1 cm	7	9		
Tumor stage			0.354	0.552
T1	13	10		
T2–4	7	8		
Metastasis			1.643	0.200
Localized	16	11		
Regional and/or distant	4	7		
Tumor classification			6.397	0.040*
NET	20	13		
NEC	0	3		
MiNEN	0	2		
Distance from the edge of the anus			0.652	0.419
≤5 cm	7	9		
>5 cm	12	9		

### 3.7 Signal pathway analysis of rectal NENs

We checked genes related to signaling pathways by using the KEGG database (gene lists of different pathways are shown in Table S4). These significantly mutated genes were related to evading apoptosis, tumor proliferation, mismatch repair, P53, TGFβ, Wnt, MAPK, PI3K/AKT, and cell cycle signaling pathways. Table 3 showed the relationship between these signaling pathways and metastatic diseases of rectal NENs. The results showed that mismatch repair (*p* = 0.030), Wnt (*p* = 0.029), MAPK (*p* = 0.016), Hedgehog (*p* = 0.037) and PI3K/AKT (*p* = 0.037) signaling pathways are involved in the metastasis of rectal NENs. Alterations in Wnt, MAPK and PI3K/AKT signaling pathways promoted metastases. The mismatch repair and Hedgehog signaling pathways were negatively correlated with metastases.

**TABLE 3:** Correlation between signaling pathway and metastasis.

Signaling pathway	Total	Localized	Regional and/or distant	*p*
Apoptosis				0.712
Mut	24	18 (47.37%)	6 (15.79%)	
Wild	14	9 (23.68%)	5 (13.16%)	
Mismatch repair				0.030
Mut	14	13 (34.21%)	1 (2.63%)	
Wild	24	14 (36.84%)	10 (26.32%)	
P53				0.322
Mut	16	10 (26.32%)	6 (15.79%)	
Wild	22	17 (44.74%)	5 (13.15%)	
Cell cycle				0.969
Mut	24	17 (44.73%)	7 (18.42%)	
Wild	14	10 (26.32%)	4 (10.53%)	
TGFβ				0.152
Mut	11	6 (15.79%)	5 (13.16%)	
Wild	27	21 (55.26%)	6 (15.79%)	
Wnt				0.029
Mut	19	10 (26.32%)	9 (23.68%)	
Wild	19	17 (44.74%)	2 (5.26%)	
MAPK				0.016
Mut	27	16 (42.10%)	11 (28.95%)	
Wild	11	11 (28,95)	0	
Hedgehog				0.037
Mut	21	18 (47.37%)	3 (7.90%)	
Wild	17	9 (23.68%)	8 (21.05%)	
PI3K/AKT				0.037
Mut	17	9 (23.68%)	8 (21.05%)	
Wild	21	18 (47.37%)	3 (7.90%)	

Table 4 showed the relationship between signaling pathways and tumor grades. Alterations in P53 (*p* = 0.009), Wnt (*p* = 0.046) and TGFβ (*p* = 0.019) signaling pathways were more pronounced in rectal NECs and MiNENs. Alterations in these signaling pathways were associated with poorly differentiated rectal NENs. In 33 cases of rectal NETG1/G2, alterations in MAPK (22 cases), Hedgehog (20 cases) and PI3K/AKT (28 cases) signaling pathways played a crucial role in development.

**TABLE 4:** Correlation between signaling pathway and tumor classification.

Signaling pathway	Total	NETG1/G2	NEC/MiNEN	*p*
Apoptosis				0.137
Mut	24	19 (50.00%)	5 (13.16%)	
Wild	14	14 (36.84%)	0	
Mismatch repair				0.633
Mut	14	13 (34.21%)	1 (2.63%)	
Wild	24	20 (52.63%)	4 (10.53%)	
P53				0.009
Mut	16	11 (28.95%)	5 (13.16%)	
Wild	22	22 (57.89%)	0	
Cell cycle				0.137
Mut	24	19 (50.00%)	5 (13.16%)	
Wild	14	14 (36.84%)	0	
Wnt signaling				0.046
Mut	19	14 (36.84%)	5 (13.16%)	
Wild	19	19 (50.00%)	0	
MAPK				0.295
Mut	27	22 (57.89%)	5 (13.16%)	
Wild	11	11 (28.95%)	0	
TGFβ				0.019
Mut	11	7 (18.42%)	4 (10.53%)	
Wild	27	26 (68.42)	1 (2.63%)	
Hedgehog				0.152
Mut	21	20 (52.63%)	1 (2.63%)	
Wild	17	13 (34.21%)	4 (10.53%)	
PI3K/AKT				1.000
Mut	33	28 (73.68%)	5 (13.16%)	
Wild	5	5 (13.16%)	0	

### 3.8 Molecular typing of rectal NENs

**FIGURE 6:**
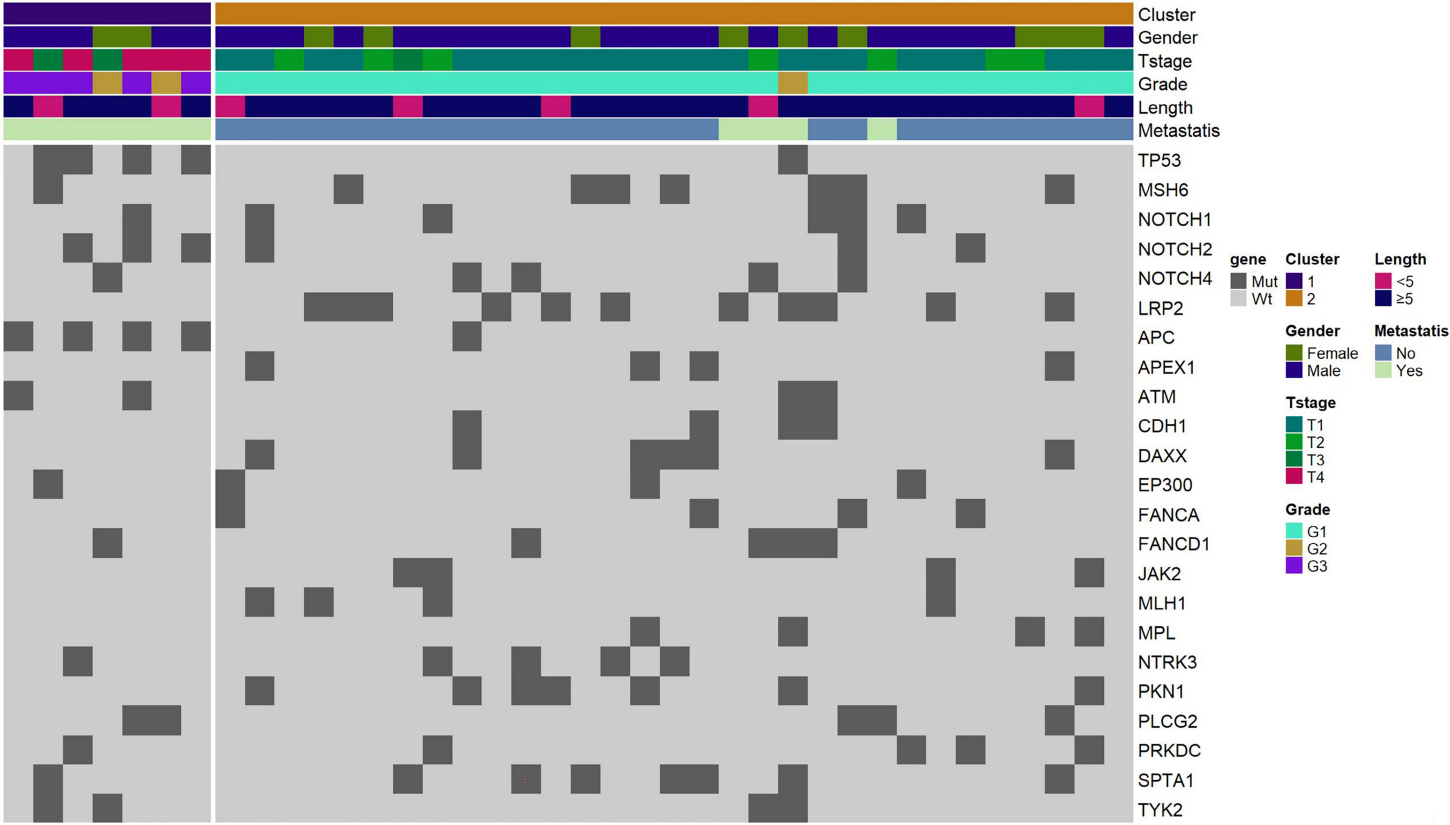
Molecular typing of rectal NEN.

Rectal NENs were classified into two molecular subtypes by cluster analysis based on the mutant genes and signaling pathways combined with clinicopathological features, named Type A and Type B (Figure 6 and Table 5). Seven patients were assigned to Type A, in which *TP53* and *APC* mutations were common, and the altered signaling pathways were Wnt and p53. Type A included NETG2 (2 cases) and NEC/MiNEN (5 cases) with advanced tumor stages. Two patients had tumor stage T3, and five had tumor stage T4. All the patients had local/distant metastases. Thirty‐one patients were assigned to Type B, in which *APEX1*, *DAXX*, *LRP2*, *NOTCH1*, *MSH6*, *SPTA1*, *CDH1*, *PKN1*, *MPL*, and *MLH1* mutations were common, and the altered signaling pathways were PI3K‐AKT, Apoptosis, Notch, and Mismatch repair. Type B included NETG1 (30 cases) and NETG2 (1 case) with early tumor stages. Twenty‐three patients had tumor stage T1, seven had tumor stage T2 and only one had tumor stage T3. Twenty‐seven patients had no metastases, and four patients had local/distant metastases. Significant differences were observed in transfer rates between the two groups (*p* = 0.000). Patients in Type B showed a trend of well‐differentiated and early‐stage tumors with less metastasis. *DAXX*, *LRP2*, and *PKN1* mutations were found only in well‐differentiated rectal NETs, which may help in distinguishing well‐differentiated and poorly differentiated rectal NENs.

**TABLE 5:** Characteristics of different molecular typing.

Molecular typing	Characteristic						
	Genes	Pathways	Grades	T‐stage	Metastatic disease		
					Metastasis	Non‐metastasis	*p*
							0.000
Cluster 1 Type A	*TP53*, *APC*	Wnt, P53	NETG2 (2 cases) NEC/MiNEN (5 cases)	T3 (2 cases) T4 (5 cases)	7 cases	0 cases	
Cluster 2 Type B	*APEX1*, *DAXX*, *LRP2*, *NOTCH1*, *MSH6*, *SPTA1*, *CDH1*, *PKN1*, *MPL*, *MLH1*	PI3K‐AKT, Apoptosis, Notch, Mismatch repair	NETG1 (30 cases) NETG2 (1 cases)	T1 (23 cases) T2 (7 cases) T3 (1 cases)	4 cases	27 cases	

## 4 DISCUSSION

As most rectal NENs are small and low‐grade, they can be discovered by colonoscopy. The risk of lymphatic metastases for small NENs (<1 cm) is 3%–10%. How to distinguish rectal NENs with high metastatic risk is therefore of important prognostic and therapeutic significance. We evaluated clinicopathological features and metastatic diseases in 62 primary rectal NENs. We observed that tumor size, tumor stage and tumor classification were associated with regional lymphatic metastases and/or distant metastases. Other studies have suggested that tumor diameter is closely associated with lymphatic or distant metastases for rectal NENs.^14^, ^15^, ^16^ This is consistent with our findings. We further stratified the risk of rectal NEN metastasis at the genetic level.

The etiology of rectal NENs is currently unknown. A previous analysis of the mutational signatures of nine colorectal NENs showed that C > T and T > C transitions are the major fraction mutations, and the verified signature showed a strong similarity to the previously described signature in COSMIC data, which correlated with the age of cancer diagnosis.^12^ In our results, eight mutational signatures were extracted, and two of them were highly matched with the COSMIC database (correlation similarity = 0.89 and 0.815), indicating that the main etiology of rectal NENs is DNA base modification and DNA mismatch repair defects; however, smoking, UV exposure and other causes also exist. Rectal NETG1 is mainly caused by DNA base modification and DNA mismatch repair defects. Besides DNA base modification and DNA mismatch repair defects, NETG2/MiNEN/NEC in the rectum is also caused by various other causes such as smoking and UV exposure. The analysis of the mutational signatures is of great significance in understanding the etiology and prevention of rectal NENs.

A recent review summarized somatic mutations in GEP‐NENs and analyzed 859 specimens collected from 820 patients from the American Association for Cancer Research (AACR) database. *ERBB2* mutations were frequently identified in well‐differentiated rectal NETs. In high‐grade rectal NECs, high mutation rates of *APC*, *KRAS*, *BRAF*, and *TP53* were found.^3^ However, the results of our study showed that the frequently mutated genes of rectal NENs are *MUC16*, *OBSCN*, *FAT1*, *ZFHX3*, *AR*, and *LRP2*. *LRP2*, *FAT3*, *PKN1*, *MERTK*, *DAXX*, *LTK*, *TET3*, *FANCD2*, and *RUNX1* mutations occurred only in rectal NETs. *APC*, *TP53*, *NF1*, *SOX9*, and *BRCA1* mutations were common in rectal NEC/MiNEN. These results were similar to those of the AACR database. A previous study showed that *PROX1* is involved in the progression of rectal NETs, and the high expression of *PROX1* is associated with metastases and poorer prognosis.^17^ Significantly different mutated genes were also found in metastatic/non‐metastatic rectal NENs in our study, and mutations in *TP53*, *TYK2*, *APC*, and *PDGFB* genes were more common in metastatic rectal NENs. *TP53* mutation was commonly seen in adenocarcinoma, such as colorectal cancer, lung adenocarcinoma and hepatocellular carcinoma, and this gene was associated with higher malignancy, and poor prognosis.^18^, ^19^ Previous studies have found that *TP53* can be used as a marker for differentiating well‐differentiated NET and NEC.^20^ *TP53* can also be used as a target to find effective drugs.

In this study, the median TMB level of rectal NENs was lower than that of other common tumors. TMB score of 47.4% of patients was ≥5 Muts/Mb, and the median TMB score of rectal NETs was 8.6 Muts/Mb. A previous study showed that the incidence of high TMB in pancreatic NETs was ≤1.5%.^21^ This was similar to our finding that NETs have a lower level of TMB. Among the 38 patients with rectal NENs in our study, all the specimens were expressed as MSS. This was similar to the findings of Tsuruoka et al's study that PD‐L1 expression was not observed in NETs.^22^ Mismatch repair protein deficiency was rare in pancreatic and intestinal NETs.^23^ In another study, microsatellite instability (MSI) was confirmed in only 3 (1.3%) of 239 NENs, and all MSI was present in cecal NECs, with MSI being uncommon in other NENs.^24^ Most of the patients in our study had rectal NETs (33 cases), and only three cases were rectal NECs. The TMB level increased with the grades,^25^ so the median TMB level of rectal NETs was lower than that of other common tumors. These data explain why TMB levels were low and all specimens were MSS and negative for PD‐L1 testing in this study. The efficacy of immunotherapy in unselected NEN populations was relatively low, especially in NETs. PD‐1 antibody therapy may be a suitable target for NECs compared with NETs.

Previous studies have shown that NENs are mainly associated with chromatin remodeling, cell cycle, PI3K/mTOR and pseudohypoxia signaling pathways.^9^, ^26^ Our results were similar to those of previous studies. However, the signaling pathways were not the same, owing to different primary sites. The results of this study showed that alterations in the Wnt, MAPK and PI3K/AKT signaling pathways promoted metastases of rectal NENs. The mismatch repair and Hedgehog signaling pathways were negatively correlated with metastases. Alterations in the p53, Wnt and TGFβ signaling pathways were associated with poorly differentiated rectal NENs. All types of GEP‐NENs had at least one potential actionable mutation that was predictive of a drug response according to the evidence levels of 1–3B in OncoKB.^27^ MEK inhibitors and mTOR/AKT/PI3K inhibitors may be effective drugs for the treatment of metastatic rectal NENs. This study showed that 81.6% of the patients with rectal NENs had DDR mutations. The most significantly mutated genes were *KMT2C*, *MSH6*, *TP53*, *FANCI*, and *BRCA*. PARP played an important role in DNA base excision repair and DNA single‐strand break repair. Patients with rectal NENs harboring DDR mutations may also benefit from PARP inhibitors. Subsequent clinical studies are needed for further confirmation.

Previous studies have made significant progress in understanding the molecular characteristics of pancreatic NETs. Four molecular subtypes have been defined, including MLP‐1 and MLP‐2, insulinoma‐like and intermediate. This has provided novel data on the immune microenvironment of pancreatic NETs.^11^ At present, no other studies have found molecular typing of rectal NENs. In this study, the sequencing results of 38 rectal NENs were analyzed. According to the mutated genes and signaling pathways, cluster typing was divided into two types: Type A and Type B. In Type A, most patients had poorly differentiated tumors, had more metastases and were at advanced stages. *TP53* and *APC* mutations were common in this type. The Wnt and p53 signaling pathways promoted metastases. However, in Type B, tumors were all well‐differentiated NETs, being at the early stage and having fewer local/distant metastases. The PI3K/AKT signaling pathway was associated with metastases in this type. *DAXX*, *LRP2*, and *PKN1* mutations occurred in only well‐differentiated rectal NETs, which may help in distinguishing well‐differentiated and poorly differentiated rectal NENs. This was similar to previous studies on pancreatic NEN. The most frequent gene alterations in pancreatic NETs were *MEN1* and *DAXX/ATRX*. In contrast, *TP53* and/or p53 proteins were identified in pancreatic NECs.^28^ These results indicate that the genetic alterations of the same type and different sites of tumors are similar, but mutated genes are different. Patients in Type A are more likely to metastasize, so the *TP53*, Wnt and p53 signaling pathways can be used as targets to explore targeted therapeutic drugs. At the same time, follow‐up time needs to be shortened during subsequent monitoring. The incidence of metastasis was low in Type B. However, *LRP2*, *FANCD1*, and *TYK2* mutations were found in two of the four patients with metastases. In terms of drug therapy, these mutated genes and the PI3K/AKT and mismatch repair signaling pathways can be further explored.

## 5 CONCLUSIONS

This study evaluated risk factors for regional lymphatic and/or distant metastases, identified high‐frequency mutated genes, mutation signatures, altered signaling pathways through NGS. Rectal NENs were divided into two molecular types. This helps to evaluate the likelihood of metastasis, formulate follow‐up strategies for patients and provide a target for future research on precision treatment of rectal NENs. PARP inhibitors, MEK inhibitors, mTOR/AKT/PI3K and Wnt signaling pathway inhibitors may be effective drugs for the treatment of metastatic rectal NENs.

## AUTHOR CONTRIBUTIONS

**Xiaoling Duan:** Data curation (equal); investigation (equal); methodology (equal); writing – original draft (equal); writing – review and editing (equal). **Man Zhao:** Investigation (equal); methodology (equal). **Xiaolei Yin:** Investigation (equal). **Lili Mi:** Investigation (equal). **Jianfei Shi:** Investigation (equal). **Ning Li:** Investigation (equal). **Xin Han:** Investigation (equal). **Guangjie Han:** Investigation (equal). **Jinfeng Wang:** Investigation (equal). **Jiaojiao Hou:** Investigation (equal). **Fei Yin:** Methodology (equal); project administration (equal); resources (equal); supervision (equal); writing – review and editing (equal).

## CONFLICT OF INTEREST STATEMENT

The authors declare no potential conflicts of interest.

## FUNDING INFORMATION

This work was supported by The Key Research And Development Plan Project of Hebei Province, Health Innovation Project (Project Number: 22377788D, Project Name: Molecular typing and precise treatment of rectal neuroendocrine neoplasms).

## Supporting information


Figure S1.

Figure S2.

Figure S3.

Figure S4.

Figure S5.

Figure S6.

Table S1.

Table S2.

Table S3.

Table S4.

## Data Availability

The data that support the findings of this study are available on request from the corresponding author. The data are not publicly available due to privacy or ethical restrictions.

## References

[1] WHO classification of Tumours Editorial Board . Digestive System Tumour. Vol 16. 5th ed. WHO Classification of Tumour; 2019.

[2] Dasari A , Shen C , Yao JC , et al. Trends in the incidence, prevalence, and survival outcomes in patients with neuroendocrine tumors in the United States. JAMA Oncol. 2017;3(10):1335‐1342. 28448665 10.1001/jamaoncol.2017.0589 PMC5824320

[3] Takayanagi D , Cho H , Machida E , et al. Update on epidemiology, diagnosis, and biomarkers in Gastroenteropancreatic neuroendocrine neoplasms. Cancers (Basel). 2022 Feb 22;14(5):1119. 35267427 10.3390/cancers14051119 PMC8909424

[4] Wang Y , Zhang Y , Lin H , et al. Risk factors for lymph node metastasis in rectal neuroendocrine tumors: a recursive partitioning analysis based on multicenter data. J Surg Oncol. 2021;124(7):1098‐1105. 34291822 10.1002/jso.26615

[5] Kudou M , Arita T , Nakanishi M , et al. Essentiality of imaging diagnostic criteria specific to rectal neuroendocrine tumors for detecting metastatic lymph nodes. Anticancer Res. 2019;39(1):505‐510. 30591502 10.21873/anticanres.13141

[6] Zhao B , Hollandsworth HM , Lopez NE , et al. Outcomes for a large cohort of patients with rectal neuroendocrine tumors: an analysis of the National Cancer Database. J Gastrointest Surg. 2021;25(2):484‐491. 32016672 10.1007/s11605-020-04525-6 PMC7396292

[7] Erstad DJ , Dasari A , Taggart MW , et al. Prognosis for poorly differentiated, high‐grade rectal neuroendocrine carcinomas. Ann Surg Oncol. 2022;29(4):2539‐2548. 34787737 10.1245/s10434-021-11016-8

[8] Heo J , Jeon SW , Jung MK , et al. A tailored approach for endoscopic treatment of small rectal neuroendocrine tumor. Surg Endosc. 2014;28:2931‐2938. 24853847 10.1007/s00464-014-3555-1

[9] Scarpa A , Chang DK , Nones K , et al. Whole‐genome landscape of pancreatic neuroendocrine tumours. Nature. 2017;543:65‐71. 28199314 10.1038/nature21063

[10] Roy S , LaFramboise WA , Liu TC . Loss of chromatin‐remodeling proteins and/or CDKN2A associates with metastasis of pancreatic neuroendocrine tumors and reduced patient survival times. Gastroenterology. 2018;154(8):2060‐2063. 29486199 10.1053/j.gastro.2018.02.026 PMC5985217

[11] Young K , Lawlor RT , Ragulan C , et al. Immune landscape, evolution, hypoxia‐mediated viral mimicry pathways and therapeutic potential in molecular subtypes of pancreatic neuroendocrine tumours. Gut. 2021;70(10):1904‐1913. 32883872 10.1136/gutjnl-2020-321016 PMC8458094

[12] Puccini A , Poorman K , Salem ME , et al. Comprehensive genomic profling of gastroenteropancreatic neuroendocrine neoplasms (GEP‐NEN). Clin Cancer Res. 2020;26(22):5943‐5951. 32883742 10.1158/1078-0432.CCR-20-1804 PMC8970533

[13] Mafcini A , Scarpa A . Genetics and epigenetics of gastroenteropancreatic neuroendocrine neoplasms. Endocr Rev. 2019;40(2):506‐536. 30657883 10.1210/er.2018-00160 PMC6534496

[14] Klempner SJ , Gershenhorn B , Tran P , Ali SM , et al. BRAFV600E mutations in high‐grade colorectal neuroendocrine tumors may predict responsiveness to BRAF‐MEK combination therapy. Cancer Discov. 2016;6(6):594‐600. 27048246 10.1158/2159-8290.CD-15-1192 PMC5008024

[15] Kim DH , Lee JH , Cha YJ , et al. Surveillance strategy for rectal neuroendocrine tumors according to recurrence risk stratification. Dig Dis Sci. 2014;59:850‐856. 24323182 10.1007/s10620-013-2972-7

[16] Kim GU , Kim KJ , Hong SM , et al. Clinical outcomes of rectal neuroendocrine tumors≤10 mm following endoscopic resection. Endoscopy. 2013;45:1018‐1023. 24288222 10.1055/s-0033-1344860

[17] Jernman J , Kallio P , Hagström J , et al. PROX1 is involved in progression of rectal neuroendocrine tumors. NETs Virchows Arch. 2015;467(3):279‐284. 26063416 10.1007/s00428-015-1795-7

[18] Salem ME , Puccini A , Grothey A , et al. Landscape of tumor mutation load, mismatch repair deficiency, and PD‐L1 expression in a large patient cohort of gastrointestinal cancers. Mol Cancer Res. 2018;16(5):805‐812. 29523759 10.1158/1541-7786.MCR-17-0735 PMC6833953

[19] Meng H , Chen G , Zhang X , et al. Stromal LRP1 in lung a denocarcinoma predicts clinical outcome. Clin Cancer Res. 2011;17(8):2426‐2433. 21325077 10.1158/1078-0432.CCR-10-2385 PMC3079007

[20] Huang XY , Shi GM , Dev Bhandari RP , et al. Low level of low‐density lipoprotein receptor‐related protein 1 predicts an unfavorable prognosis of hepatocellular carcinoma after curative resection. PLoS One. 2012;7(3):e32775. 22427881 10.1371/journal.pone.0032775 PMC3299691

[21] Derks JL , Rijnsburger N , Hermans BCM , et al. Clinical‐pathologic challenges in the classification of pulmonary neuroendocrine neoplasms and targets on the horizon for future clinical practice. J Thorac Oncol. 2021;16(10):1632‐1646. 34139363 10.1016/j.jtho.2021.05.020

[22] Tsuruoka K , Horinouchi H , Goto Y , et al. PD‐L1 expression in neuroendocrine tumors of the lung. Lung Cancer. 2017;108:115‐120. 28625622 10.1016/j.lungcan.2017.03.006

[23] Prisciandaro M , Antista M , Raimondi A , et al. Biomarker landscape in neuroendocrine tumors with high‐grade features: current knowledge and future perspective. Front Oncol. 2022;12:780716. 35186729 10.3389/fonc.2022.780716 PMC8856722

[24] Fraune C , Simon R , Hube‐Magg C , et al. Homogeneous MMR deficiency throughout the entire tumor mass occurs in a subset of colorectal neuroendocrine carcinomas. Endocr Pathol. 2020;31(2):182‐189. 32144630 10.1007/s12022-020-09612-7 PMC7250944

[25] Mitsuhashi K , Yamamoto I , Kurihara H , et al. Analysis of the molecular features of rectal carcinoid tumors to identify new biomarkers that predict biological malignancy. Oncotarget. 2015;6(26):22114‐22125. 26090613 10.18632/oncotarget.4294 PMC4673150

[26] Szybowska M , Mete O , Weber E , Silver J , Kim RH . Neuroendocrine neoplasms associated with germline pathogenic variants in the homologous recombination pathway. Endocr Pathol. 2019;30(3):237‐245. 30772928 10.1007/s12022-019-9569-4

[27] Chakravarty D , Gao J , Phillips SM , et al. OncoKB: a precision oncology Knowledge Base. JCO Precis Oncologia. 2017. doi:10.1200/PO.17.00011 PMC5586540 28890946

[28] Konukiewitz B , Jesinghaus M , Steiger K , et al. Pancreatic neuroendocrine carcinomas reveal a closer relationship to ductal adenocarcinomas than to neuroendocrine tumors G3. Hum Pathol. 2018;77:70‐79. 29596894 10.1016/j.humpath.2018.03.018

