# Supplementary material for: Molecular typing and mutational characterization of rectal neuroendocrine neoplasms
Source: Cancer Med. 2023 Jun 30;12(15):16207–20. doi: 10.1002/cam4.6281 (PMC10469650; doi:10.1002/cam4.6281)
Supplement: Supplementary file 1 — Figure S1. [file CAM4-12-16207-s010.doc]

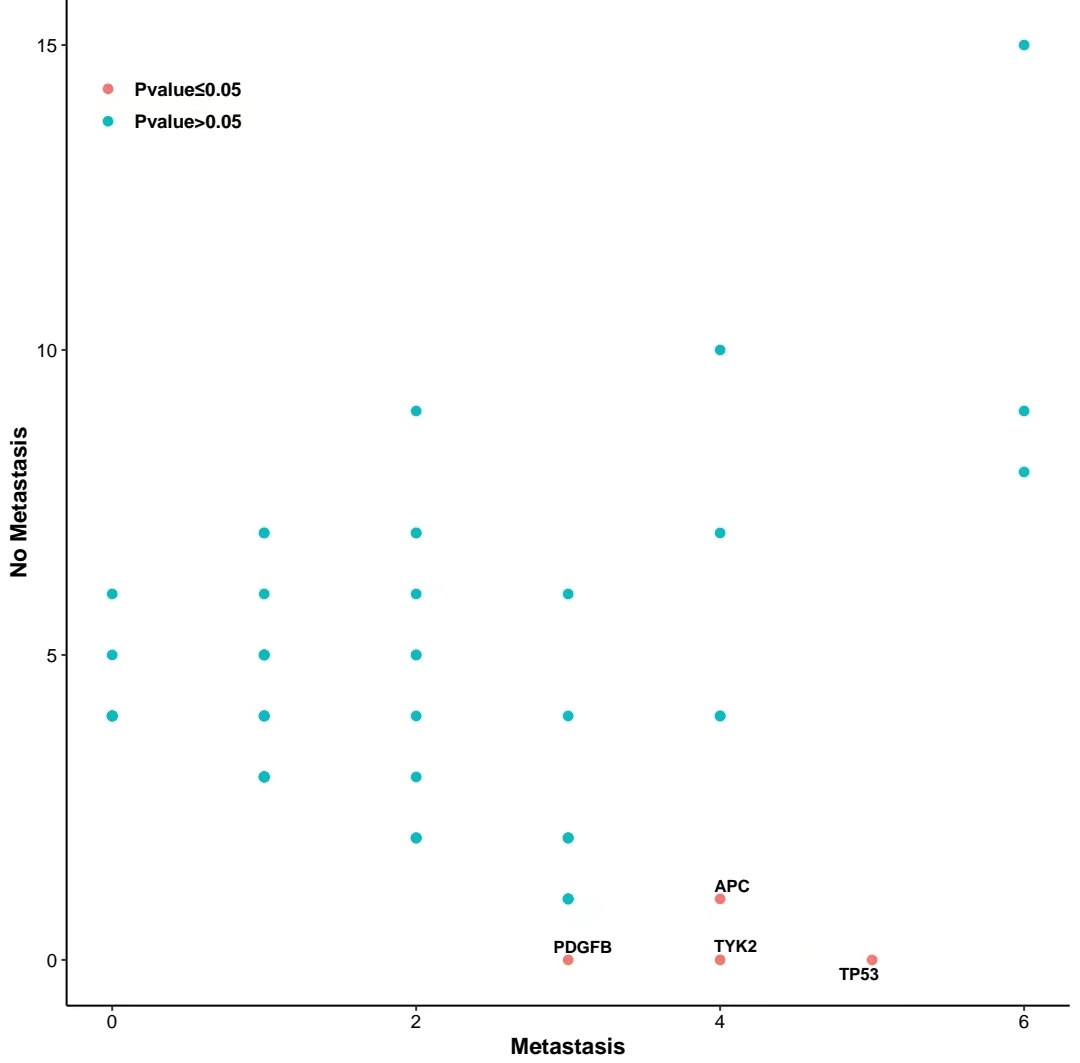


Figure S1 Scatter plots depicting the mutational frequencies (percentage of patients) between patients with metastases and without metastases in our cohort. Each dot represents one gene, and dots are color coded according to the P-values (-log10(P) uncorrected) shown in the legend. Statistics shown were derived from two-sided Fisher’s exact tests.
