# Supplementary material for: Molecular typing and mutational characterization of rectal neuroendocrine neoplasms
Source: Cancer Med. 2023 Jun 30;12(15):16207–20. doi: 10.1002/cam4.6281 (PMC10469650; doi:10.1002/cam4.6281)
Supplement: Supplementary file 2 — Figure S2. [file CAM4-12-16207-s008.doc]

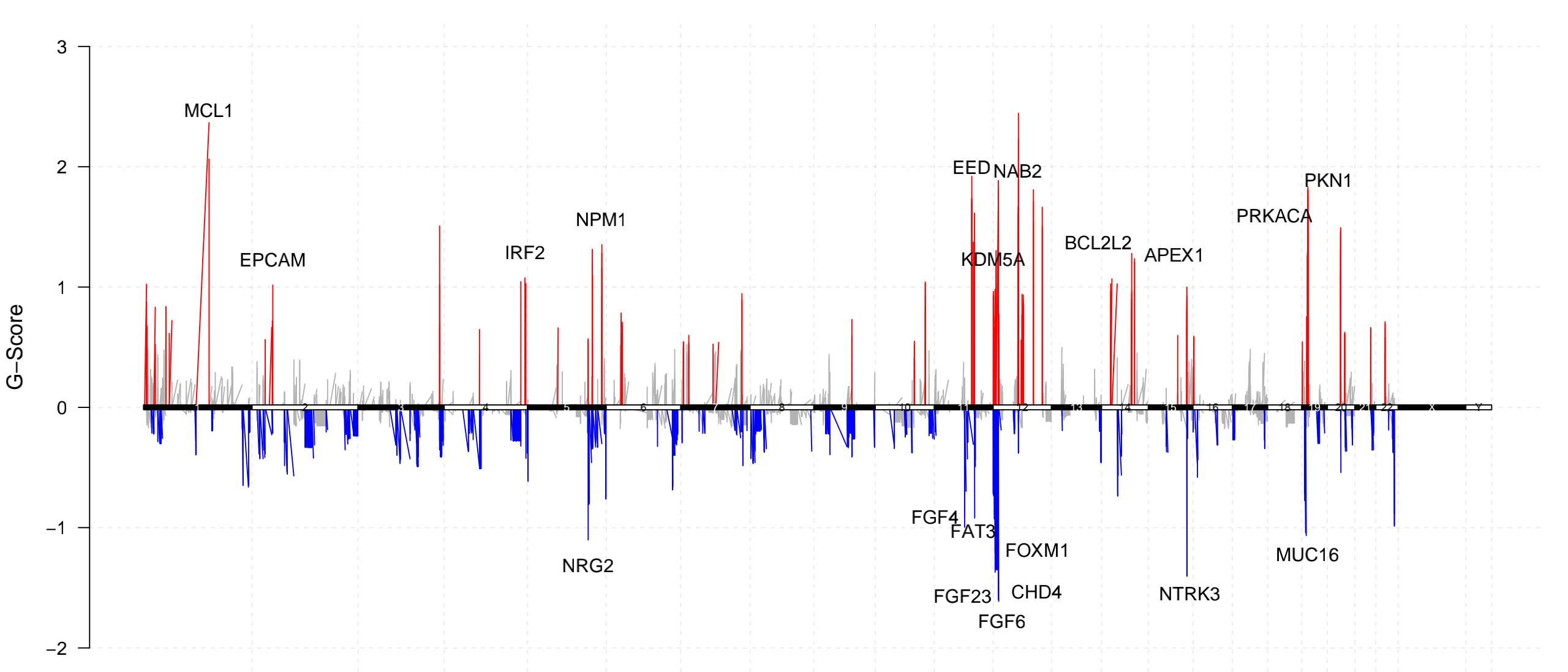


Figure S2 Significant (q < 0.1) focal somatic copy-number alterations (SCNAs) along all chromosomes. The vertical axis indicates the G-scores generated from GISTIC2, which considers the amplitude of the aberration and the frequency of its occurrence across samples. Recurrent SCNAs of top 20 mutated genes were also highlighted.
