# Supplementary material for: Molecular typing and mutational characterization of rectal neuroendocrine neoplasms
Source: Cancer Med. 2023 Jun 30;12(15):16207–20. doi: 10.1002/cam4.6281 (PMC10469650; doi:10.1002/cam4.6281)
Supplement: Supplementary file 3 — Figure S3. [file CAM4-12-16207-s003.doc]

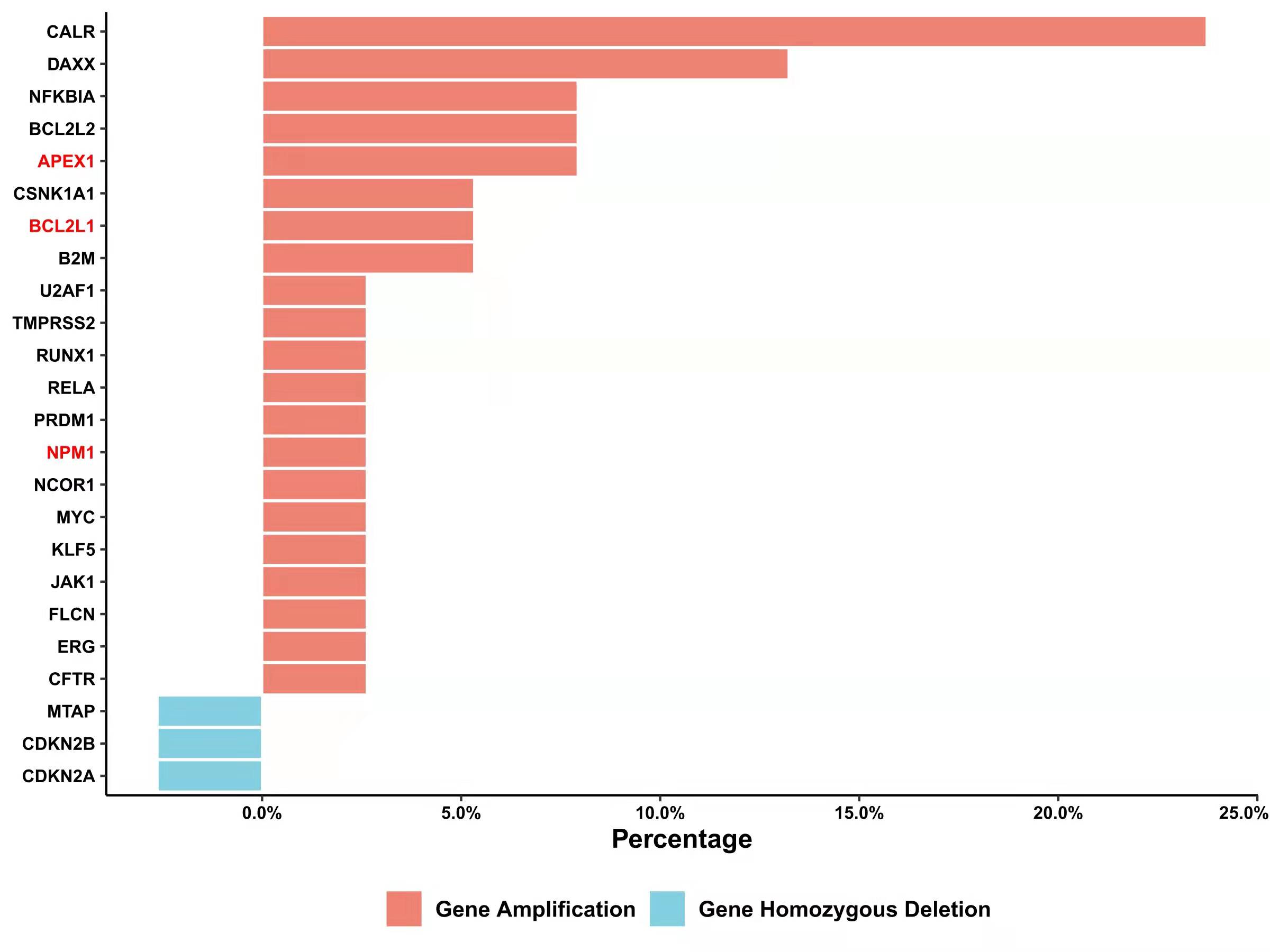


Figure S3 Proportions of patients from the entire cohort for with significantly amplified (red) or deleted (blue) cancer driver genes.
