# Supplementary figures and images for: Molecular typing and mutational characterization of rectal neuroendocrine neoplasms
Source: Cancer Med. 2023 Jun 30;12(15):16207–20. doi: 10.1002/cam4.6281 (PMC10469650; doi:10.1002/cam4.6281)

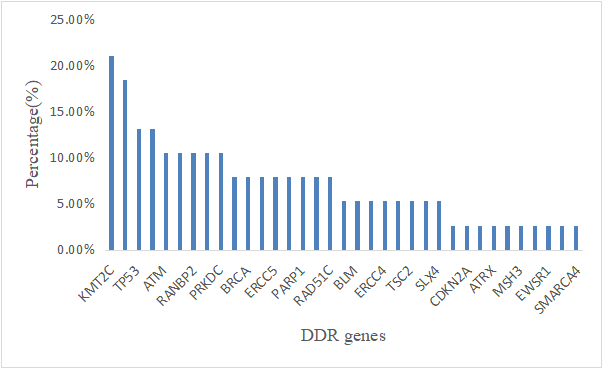


Figure S4 DDR mutant genes in rectal NENs

Supplement: Supplementary file 4 — Figure S4. [file CAM4-12-16207-s004.doc]

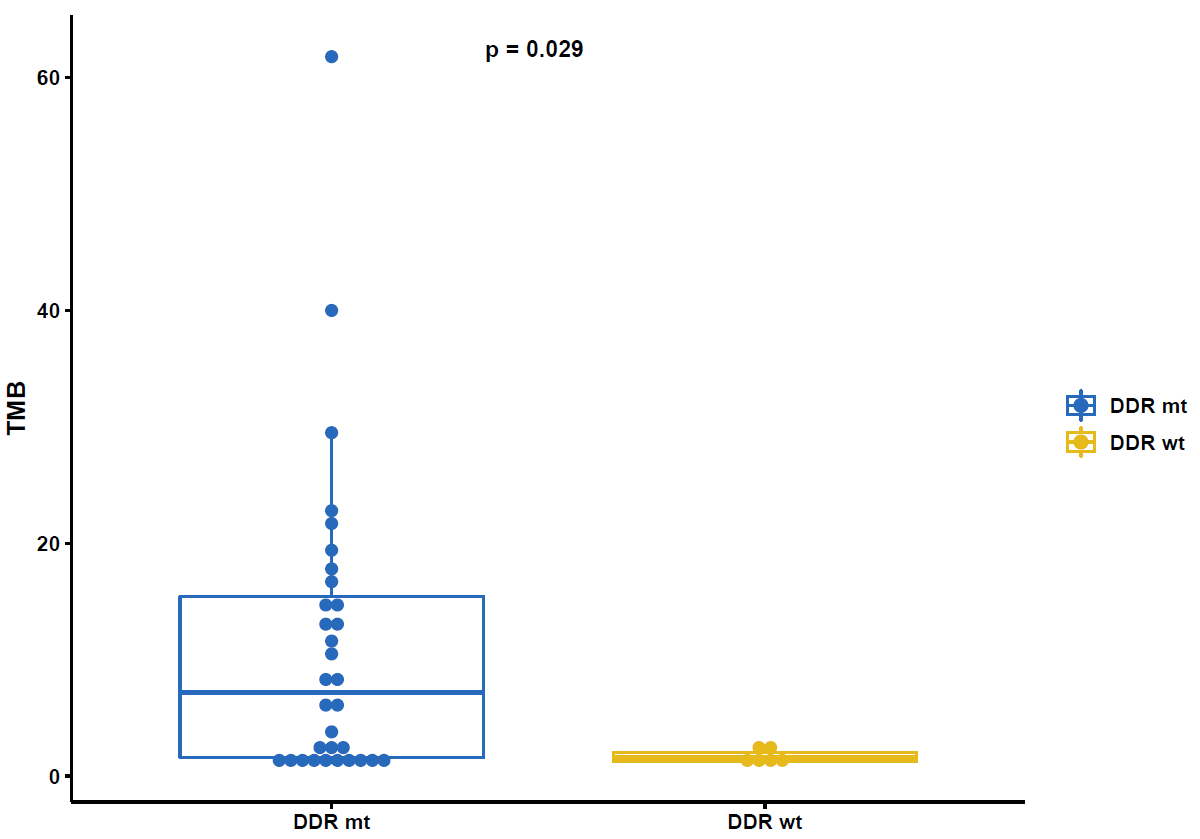


Figure S5 Comparisons of TMB between DDR mutant and wild-type DDR genes.

Supplement: Supplementary file 5 — Figure S5. [file CAM4-12-16207-s005.doc]

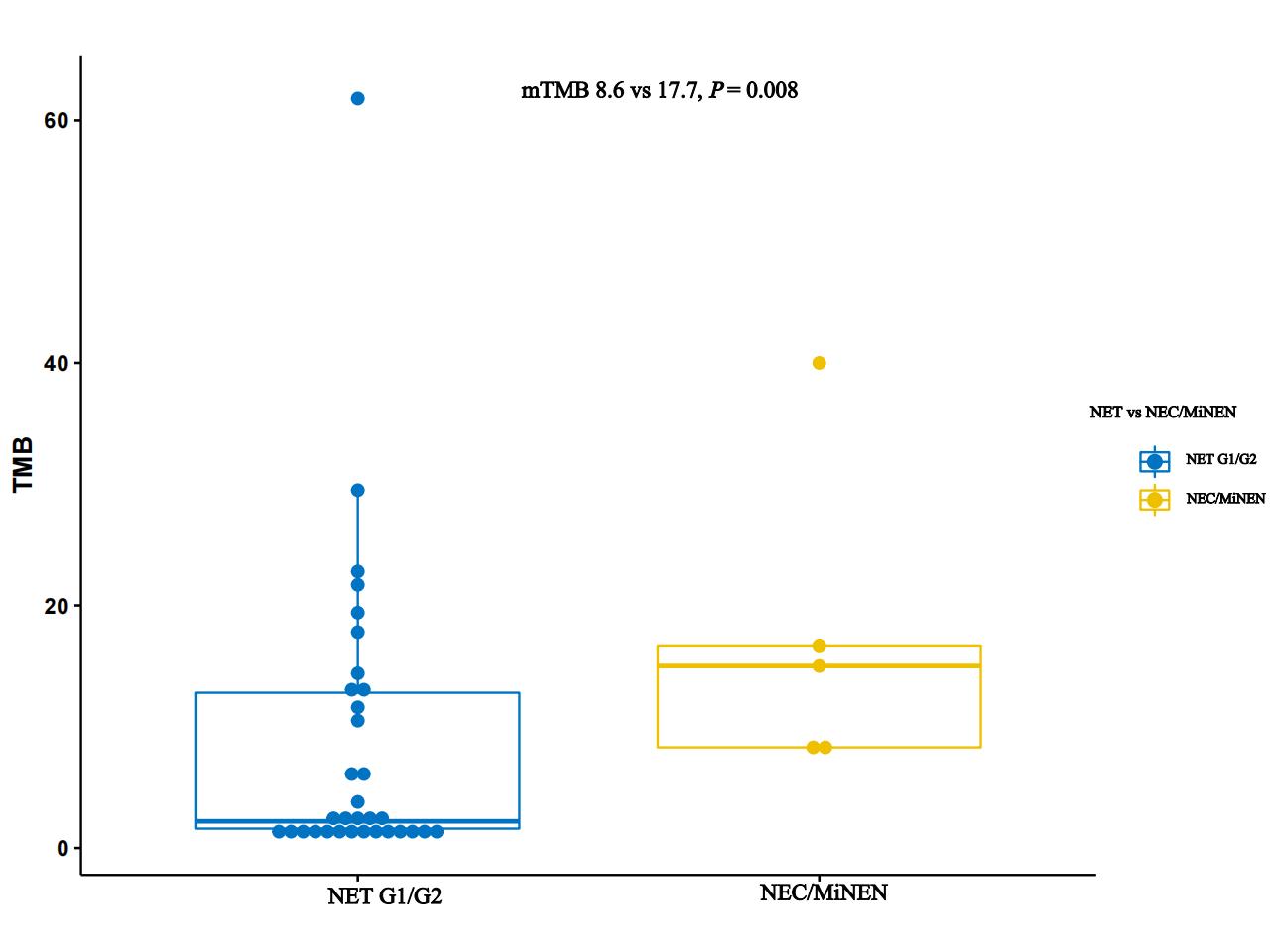
Figure S6 Relationship between TMB and tumor classification

Supplement: Supplementary file 6 — Figure S6. [file CAM4-12-16207-s006.doc]
