# Supplementary material for: Molecular typing and mutational characterization of rectal neuroendocrine neoplasms
Source: Cancer Med. 2023 Jun 30;12(15):16207–20. doi: 10.1002/cam4.6281 (PMC10469650; doi:10.1002/cam4.6281)
Supplement: Supplementary file 7 — Table S1. [file CAM4-12-16207-s002.doc]

Table S1 Differential mutated genes in different grades of RNEN

| Gene | Mutation_NETG1/G2 | Mutation_NEC+MiNEN | Wildtype_  NETG1/G2 | Wildtype_  NEC+MiNEN | *P* |
| --- | --- | --- | --- | --- | --- |
| *APC* | 1 | 4 | 32 | 1 | <0.001 |
| *TP53* | 1 | 4 | 32 | 1 | <0.001 |
| *FPR1* | 1 | 3 | 32 | 2 | 0.005 |
| *FAT4* | 2 | 3 | 31 | 2 | 0.011 |
| *NOTCH2* | 2 | 3 | 31 | 2 | 0.011 |
| *BRCA1* | 0 | 2 | 33 | 3 | 0.014 |
| *FBXW7* | 0 | 2 | 33 | 3 | 0.014 |
| *NF1* | 0 | 2 | 33 | 3 | 0.014 |
| *SOX9* | 0 | 2 | 33 | 3 | 0.014 |
| *ANGPT2* | 1 | 2 | 32 | 3 | 0.040 |
| *KEL* | 1 | 2 | 32 | 3 | 0.040 |
| *NOTCH3* | 1 | 2 | 32 | 3 | 0.040 |
| *PDGFB* | 1 | 2 | 32 | 3 | 0.040 |
| *RPTOR* | 1 | 2 | 32 | 3 | 0.040 |
| *ATM* | 2 | 2 | 31 | 3 | 0.076 |
| *HDAC9* | 2 | 2 | 31 | 3 | 0.076 |
| *PTPRT* | 2 | 2 | 31 | 3 | 0.076 |
| *LRP2* | 11 | 0 | 22 | 5 | 0.295 |
| *FAT3* | 9 | 0 | 24 | 5 | 0.312 |
| *PKN1* | 7 | 0 | 26 | 5 | 0.561 |
| *DAXX* | 6 | 0 | 27 | 5 | 0.570 |
| *LTK* | 6 | 0 | 27 | 5 | 0.570 |
| *MERTK* | 6 | 0 | 27 | 5 | 0.570 |
| *CALR* | 7 | 2 | 26 | 3 | 0.574 |
| *LRP1B* | 7 | 2 | 26 | 3 | 0.574 |
| *AR* | 9 | 2 | 24 | 3 | 0.615 |
| *FAT1* | 13 | 1 | 20 | 4 | 0.633 |
| *MUC16* | 18 | 3 | 15 | 2 | 1.000 |
| *OBSCN* | 13 | 2 | 20 | 3 | 1.000 |
| *ZFHX3* | 12 | 2 | 21 | 3 | 1.000 |
| *ADGRA2* | 7 | 1 | 26 | 4 | 1.000 |
| *ARID1B* | 7 | 1 | 26 | 4 | 1.000 |
| *KMT2C* | 7 | 1 | 26 | 4 | 1.000 |
| *MSH6* | 7 | 1 | 26 | 4 | 1.000 |
| *SPTA1* | 7 | 1 | 26 | 4 | 1.000 |
| *KEAP1* | 6 | 1 | 27 | 4 | 1.000 |
| *TET1* | 6 | 1 | 27 | 4 | 1.000 |
| *NOTCH1* | 5 | 1 | 28 | 4 | 1.000 |
| *PTPRO* | 5 | 1 | 28 | 4 | 1.000 |
| *TRAF7* | 5 | 1 | 28 | 4 | 1.000 |
| *FANCD2* | 5 | 0 | 28 | 5 | 1.000 |
| *RUNX1* | 5 | 0 | 28 | 5 | 1.000 |
| *TET3* | 5 | 0 | 28 | 5 | 1.000 |

MT：mutation type；WT：wild type
