# Supplementary material for: Molecular typing and mutational characterization of rectal neuroendocrine neoplasms
Source: Cancer Med. 2023 Jun 30;12(15):16207–20. doi: 10.1002/cam4.6281 (PMC10469650; doi:10.1002/cam4.6281)
Supplement: Supplementary file 8 — Table S2. [file CAM4-12-16207-s007.doc]

Table S2 Different mutant genes in metastatic/non-metastatic RNEN

| Gene | MT_non | MT_metastasis | WT_non | WT_metastasis | P value |
| --- | --- | --- | --- | --- | --- |
| *TP53* | 0 | 5 | 27 | 6 | 0.001 |
| *TYK2* | 0 | 4 | 27 | 7 | 0.004 |
| *APC* | 1 | 4 | 26 | 7 | 0.019 |
| *PDGFB* | 0 | 3 | 27 | 8 | 0.020 |
| *ATM* | 1 | 3 | 26 | 8 | 0.065 |
| *FPR1* | 1 | 3 | 26 | 8 | 0.065 |
| *BRCA1* | 0 | 2 | 27 | 9 | 0.078 |
| *FBXW7* | 0 | 2 | 27 | 9 | 0.078 |
| *NF1* | 0 | 2 | 27 | 9 | 0.078 |
| *SOX9* | 0 | 2 | 27 | 9 | 0.078 |
| *SPEN* | 0 | 2 | 27 | 9 | 0.078 |
| *FAT4* | 2 | 3 | 25 | 8 | 0.134 |
| *DAXX* | 6 | 0 | 21 | 11 | 0.154 |
| *ZFHX3* | 8 | 6 | 19 | 5 | 0.266 |
| *OBSCN* | 9 | 6 | 18 | 5 | 0.285 |
| *FAT1* | 10 | 4 | 17 | 7 | 1.000 |
| *MUC16* | 15 | 6 | 12 | 5 | 1.000 |

MT-non：mutation type - non-metastasis.

WT-non：wild type - non-metastasis.
