# Supplementary material for: Molecular typing and mutational characterization of rectal neuroendocrine neoplasms
Source: Cancer Med. 2023 Jun 30;12(15):16207–20. doi: 10.1002/cam4.6281 (PMC10469650; doi:10.1002/cam4.6281)
Supplement: Supplementary file 9 — Table S3. [file CAM4-12-16207-s009.doc]

Table S3 Relationship between CNV and clinicopathological

| Factors | None | Amplification/  Deletion | *P* |
| --- | --- | --- | --- |
| Gender |  |  | 1.000 |
| Male | 7 | 20 |  |
| Female | 3 | 8 |  |
| Age |  |  | 0.460 |
| ≤55y | 3 | 14 |  |
| >55y | 7 | 14 |  |
| Tumor size |  |  | 0.267 |
| ≤1cm | 4 | 18 |  |
| >1cm | 6 | 10 |  |
| Tumor stage |  |  | 0.473 |
| T1 | 5 | 18 |  |
| T2-4 | 5 | 10 |  |
| Metastasis |  |  | 0.224 |
| Localized | 9 | 17 |  |
| Regional and/or distant | 1 | 10 |  |
| Tumor classification |  |  | 0.358 |
| NET | 10 | 23 |  |
| NEC | 0 | 3 |  |
| MiNEN | 0 | 2 |  |
| distance from the edge of the anus |  |  | 0.274 |
| ≤5cm | 6 | 10 |  |
| >5cm | 4 | 17 |  |
