# Supplementary material for: Molecular typing and mutational characterization of rectal neuroendocrine neoplasms
Source: Cancer Med. 2023 Jun 30;12(15):16207–20. doi: 10.1002/cam4.6281 (PMC10469650; doi:10.1002/cam4.6281)
Supplement: Supplementary file 10 — Table S4. [file CAM4-12-16207-s001.doc]

Table S4 Key mutated genes and pathways identified in rectal NEN

| Signaling Pathway | Genes |
| --- | --- |
| Apoptosis | *SPTA1, DAXX, TP53, ATM, NFKBIA, PARP1, RELA, BCL2L1, KRAS, PARP4, PIK3R1, AKT1, MAP2K2, MCL1, NTRK1, PARP3, PIK3CB, PIK3CD, RAF1* |
| Mismatch repair | *MLH1, MSH3, MSH6, PMS2, POLP1* |
| P53 signaling | *TP53, ATM, ATR, BCL2L1, CCNE1, CHEK2, TSC2, CDKN1A, CDKN2A* |
| Cell cycle | *PRKDC, TP53, ATM, EP300, ATR, CREBBP, CCNE1*  *CHEK2, RB1, SMAD4, ABL1, CDKN1A, CDKN1B, CDKN2A, CDKN2B, HDAC1, MYC, WEE2, YWHAE* |
| TGFβsignaling | *EP300, CREBBP, SMAD4, ACVR1B, CDKN2B, INHBA, MYC* |
| Wnt signaling | *APC, TP53, EP300, CREBBP, CSNK1A1, SMAD4, AXIN1, AXIN2, CTNNB1, MYC, PRKACA, RAC1, RNF43, ROCK2* |
| MAPK signaling | *DAXX, TP53, KDR, MET, PDGFB, RELA, ERBB4, FGFR2, FLT4, KRAS, NF1, PDGFRB, RELB, VEGFA, AKT1, CSF1R, EGFR, EPHA2, ERBB3, FGF2, FGF21, FGF23, FGF4, FGF6, FGFR1, FGFR3, FGFR4, FLT1, MAP2K2, MAP2K4, MECOM, MKNK1, MYC, NTRK1, PRKACA, RAC1, RAF1* |
| Hedgehog signaling | *LRP2, GLI2, SMO, CSNK1A1, GLI1, GLI3, PTCH1, PRKACA, SPOP* |
| PI3K/AKT | *EGF, PDGF, FGF, MET, RAS, AKT* |
